# Green Synthesis of Magnesium Nitrate Nanoparticles Using *Momordica charantia* Peel Extract: Enhanced Antibacterial Activity and Antibiotic Potentiation Against Multidrug-Resistant Bacteria with Low Acute Toxicity

**DOI:** 10.3390/nano16120728

**Published:** 2026-06-12

**Authors:** Iffat Naz, Humaira Niaz, Abdul Rehman, Mubbashir Hussain, Imen Ben Abdelmalek, Malik Nawaz Shuja, Muhammad Anees

**Affiliations:** 1 Department of Biology, College of Science, Qassim University, Buraydah 51452, Saudi Arabia; mm.abdulmalek@qu.edu.sa; 2 Department of Microbiology, Kohat University of Science and Technology (KUST), Kohat 26000, Khyber Pakhtunkhwa, Pakistan; humairaniaz59@gmail.com (H.N.); mubbashir.hussain@kust.edu.pk (M.H.); maliknshuja@gmail.com (M.N.S.); dr.anees@kust.edu.pk (M.A.)

**Keywords:** *Momordica charantia* peel, nanoparticles, magnesium nitrate nanoparticles, multidrug-resistant bacteria, antibacterial activity, toxicity assessment

## Abstract

Multidrug-resistant bacterial pathogens pose a critical global health challenge, necessitating safe and effective antimicrobial alternatives. Plant-derived nanoparticles represent promising candidates due to their bioactivity and biocompatibility. Magnesium nitrate nanoparticles were synthesized using *Momordica charantia* peel extract through green chemistry. Phytochemical screening identified flavonoids, phenolics, tannins, and terpenoids that facilitated nanoparticle formation and stability. Characterization via scanning electron microscopy, energy-dispersive X-ray spectroscopy, X-ray diffraction, and Fourier transform infrared spectroscopy confirmed polydisperse size distribution (1–100 nm), crystalline structure, and functional group capping. Disc diffusion assays demonstrated concentration-dependent antibacterial activity against multidrug-resistant strains, with maximum inhibition zones of 17.6 ± 1.1 mm against Gram-positive bacteria. Minimum inhibitory concentration and minimum bactericidal concentration assays revealed high bactericidal activity, particularly against Gram-positive bacteria. Time-kill kinetic studies showed concentration- and time-dependent killing with ≥3 log_10_ reduction in viable bacterial counts at higher concentrations. Nanoparticle–antibiotic combinations exhibited markedly enhanced activity against multidrug-resistant strains compared to free antibiotics, indicating synergistic effects. Toxicity assessment using Brine Shrimp Lethality Assay revealed low toxicity (LC_50_ > 1000 µg/mL). Green-synthesized magnesium nitrate nanoparticles demonstrate potent antibacterial properties and effectively enhance antibiotic potency against multidrug-resistant bacteria. Further studies are required to validate therapeutic applicability.

## 1. Introduction

Antibiotic resistance (ABR) is a pressing global issue that has compromised the effectiveness of traditional antibiotic treatment strategies and contributed to rising mortality, morbidity and economic burdens [1,2]. The emergence and spread of multidrug-resistant (MDR) bacteria, which are resistant to multiple antibiotics, have made this problem even worse and have triggered the need for new and sustainable approaches to alternative therapy [2].

MDR infections are a significant public health crisis worldwide. Epidemiological evidence suggests that MDR bacterial infections are a major cause of morbidity and mortality in the world, and the costs of treatment are growing significantly [3,4]. People with MDR infections suffer significantly longer hospital stays, higher treatment expenses and higher death rates than people with susceptible infections [1,2]. The three primary MDR pathogens addressed in this study, such as *Staphylococcus aureus*, *Escherichia coli*, and *Pseudomonas* spp., are among the most pathologically relevant microorganisms causing diverse clinical infections [5,6]. *S. aureus* is responsible for serious life-threatening skin and soft tissue infections, hospital-acquired pneumonia, bacteremia and endocarditis; methicillin-resistant *S. aureus* (MRSA) is responsible for much of the hospital-acquired infection problem worldwide. *E. coli* is a major cause of urinary tract infections, sepsis, and meningitis, and ESBL-producing strains are becoming more common in clinical practice. *Pseudomonas aeruginosa* is responsible for chronic respiratory infections in hospitalized and immunocompromised patients, wound infections in burn patients, and chronic lung infections in patients with Cystic Fibrosis. The resistance mechanisms, such as drug inactivation, alteration of drug targets, reduced membrane permeability, and efflux pumps [4], make multiple classes of antibiotics ineffective for the treatment of these infections, making the need for alternative antimicrobial strategies a pressing priority as advocated by the World Health Organization [7].

In this regard, nanotechnology has emerged as an interdisciplinary technology to tackle the issue of antimicrobial resistance. Controlling the manipulation of matter at the nanoscale provides distinct physicochemical properties such as high surface area, increased reactivity and enhanced cellular uptake, which contribute to enhanced antimicrobial properties [8,9]. Nanoparticles can interact with microbial cells in several ways, including cell wall destruction, generation of reactive oxygen species (ROS) and disruption of key cellular processes, thereby reducing the resistance and MDR phenomenon [10]. Additionally, the combination of nanotechnology and biology has led to the emergence of green nanotechnology with an emphasis on environmentally, economically and sustainably safe methods of synthesis. Plant-mediated biosynthesis of nanoparticles has gained considerable attention due to its simplicity, non-toxicity and absence of toxic chemicals, and offers a safe alternative to conventional physicochemical strategies [11,12,13].

*Momordica charantia* (bitter gourd) is an important bioresource among plants with a range of phytochemicals and medicinal properties. *M. charantia* has been traditionally used as an antioxidant, anti-diabetic, anti-inflammatory and antimicrobial agent [14]. It contains a range of phytoconstituents like flavonoids, phenolic acids, alkaloids and proteins like momordin that are responsible for its therapeutic properties [15,16]. These phytoconstituents play a crucial role in the green synthesis of nanoparticles, acting as reducing and capping agents, which affect their size, shape, and activity. Using *M. charantia* extract for nanoparticle synthesis not only improves biocompatibility but also offers potential synergistic antimicrobial properties.

The current study aims to fill the existing gaps in the evaluation of nanoparticle-based antimicrobials using qualitative, quantitative, and safety assessments. Although qualitative zone of inhibition methods have been commonly used in previous studies, such methods fail to offer accurate information on antimicrobial and bactericidal activity. Thus, alongside traditional disc diffusion assays, this study includes minimum inhibitory concentration (MIC), minimum bactericidal concentration (MBC) and growth rate analyses to allow a more quantitative assessment of antimicrobial activity. While magnesium nitrate nanoparticles [Mg(NO_3_)_2_-NPs] hold the promise of effective antimicrobials, little is known about their toxicity, even in preliminary biological systems. To overcome this concern, the current study evaluated Mg(NO_3_)_2_-NPs toxicity using the Brine Shrimp Lethality test as a fast and efficient model for biocompatibility. Therefore, the present study intends to (i) prepare Mg(NO_3_)_2_-NPs using *M. charantia* peel extract, (ii) assess its antibacterial activity against MDR bacterial pathogens using qualitative and quantitative approaches, (iii) determine its synergism with conventional antibiotics, and (iv) assess its preliminary brine shrimp toxicity. This interdisciplinary approach gives more insight into the medicinal properties and safety of eco-friendly nanoparticles.

## 2. Materials and Methods

### 2.1. Collection and Identification of MDR Bacterial Isolates

Previously identified bacterial isolates, such as *E. coli*, *S. aureus* and *Pseudomonas* spp., were collected from the culture bank of the Microbiology Research Laboratory (MRL), Kohat University of Science and Technology (KUST), Kohat. The isolates were reactivated and sub-cultured on nutrient agar medium before being used. Bacterial identification was confirmed by Gram staining and standard biochemical tests (oxidase, catalase, indole, citrate and triple sugar iron (TSI) tests) following standard microbiological techniques [17]. The MDR nature of the isolates was confirmed by antibiotic susceptibility tests, as per the guidelines of the Clinical and Laboratory Standards Institute [18].

### 2.2. Plant Material and Extract Preparation

The fruits of *M. charantia* were obtained from the local market in Jand, District Attock, Punjab, Pakistan. The fruit was washed with distilled water, sliced and dried in the shade for 5–6 days. The dried powder was pulverized into a fine powder with the help of a blender and stored in airtight containers at room temperature for future use.

#### 2.2.1. Aqueous Extract of *M. charantia*

The aqueous extract of *M. charantia* was obtained by adding 25 g of powdered plant to 100 mL of distilled water. The suspension was heated for 1 h at approximately 80–90 °C with frequent shaking for the extraction of its therapeutic components. It was cooled to room temperature and filtered using Whatman No. 1 filter paper. This was then evaporated to semi-concentrated form (50 °C) using a rotary evaporator and was used for the preparation of nanoparticles and antibacterial activity [19,20].

#### 2.2.2. Methanolic Extract of *M. charantia*

The methanolic extract of *M. charantia* was prepared by extracting 25 g of powdered plant sample with 150 mL of methanol and left at room temperature for 24 h with occasional shaking. The mixture was filtered through Whatman filter paper, and the solvent was evaporated in a rotary evaporator under reduced pressure (40 °C) to get a concentrated crude extract. The concentrated extract was dried at 50 °C in a hot air oven to remove traces of solvent and stored for analysis [19,20].

### 2.3. Phytochemical Analysis of M. charantia Extracts

The qualitative phytochemical analysis of aqueous and methanolic extracts was carried out to identify the major bioactive compounds present in them. Different bioactive compounds such as saponins, phenolics, flavonoids, tannins, and terpenoids were screened using standard colourimetric and precipitation-based methods as described by Harith et al. [21] by looking for color change and/or froth formation.

### 2.4. Antibacterial Activity of M. charantia Extracts

Antibacterial activity of *M. charantia* was tested by the agar well diffusion method [22]. Bacterial suspensions subjected to standard turbidity were spread on the surface of Mueller–Hinton agar (MHA) plates. Wells of approximately 6 mm diameter were bored in the agar, and 80 µL of aqueous and methanolic extracts were added to each well. Dimethyl sulfoxide (DMSO) was used as a negative control, and antibiotic discs as a positive control. The plates were incubated at 37 °C for 24 h, and zones of inhibition were measured in millimetres.

### 2.5. Green Synthesis of Mg(NO_3_)_2_-NPs

Mg(NO_3_)_2_-NPs were prepared by a green synthesis method using *M. charantia* peel extract. About 2 g of magnesium nitrate was mixed with 100 mL of deionized water. The extract was added slowly while stirring with a magnetic stirrer. The pH of the reaction solution was maintained at around 10 by the gradual addition of NaOH. The mixture was then stirred for 2 h until the appearance of a whitish-yellow precipitate, which indicated the formation of nanoparticles. The solution was then centrifuged at 4000 rpm for 15 min, and the precipitate was washed with distilled water. The nanoparticles were then dried at 80 °C and stored for future use.

### 2.6. Characterization of Mg(NO_3_)_2_-NPs

Scanning electron microscopy (SEM), energy-dispersive X-ray spectroscopy (EDAX), X-ray diffraction (XRD), and Fourier transform infrared spectroscopy (FTIR) were carried out at the Centralized Resource Laboratory (CRL) and National Centre of Excellence in Geology (NCEG), University of Peshawar, to characterize the synthesized Mg(NO_3_)_2_-NPs. For SEM analysis, a small amount of dried nanoparticle powder was spread uniformly on a carbon-coated aluminum stub. The stubs were gold-coated using a vacuum sputter coater to improve sample conductivity and prevent charging artefacts. The sputter-coated samples were then analyzed by SEM at accelerating voltages ranging from 10 to 20 kV. Images were taken to study the structure, size and dispersion of the nanoparticles.

EDAX was also conducted along with SEM to identify the elemental composition of the samples. In SEM, the bombardment of the sample by the incident electron beam resulted in the emission of X-rays. These emissions were captured and used to confirm the presence of magnesium and other elements, and the weight percentage of these elements was determined from the EDAX spectra.

In the XRD analysis, the dried nanoparticle sample was ground and prepared on a sample holder with a flat surface. Further, an X-ray diffractometer with Cu-Kα radiation (λ = 1.5406 Å) was used. The X-ray diffraction data were collected over a 2θ range of around 10° to 80° at a rate of around 2°/min. The diffraction peaks were matched with reference patterns of the Joint Committee on Powder Diffraction Standards (JCPDS database) to establish the crystallinity, purity and average size of the nanoparticles.

FTIR analysis was employed to study the functional groups on the nanoparticle surface and the involvement of biomolecules of the plant extract in the synthesis and stability of the nanoparticles. The spectra were recorded using the KBr pellet method, where a small quantity of the dried nanoparticles was mixed with KBr powder and pressed into a pellet. The measurements were taken between 4000 and 400 cm^−1^. The absorption peaks were used to determine the presence of functional groups responsible for the reduction and capping of nanoparticles.

### 2.7. Antibiotics Used in the Study

Antibiotics used in this study were erythromycin (5 µg/disc), ceftazidime (30 µg/disc), penicillin (15 µg/disc) and oxacillin (5 µg/disc). Antibiotic susceptibility was tested following the CLSI [18] guidelines.

### 2.8. Preparation of Mg(NO_3_)_2_-NPs Coated Filter Paper Discs

Sterile filter paper discs (about 6 mm diameter) were made from Whatman No. 1 filter paper by autoclaving at 121 °C for 15 min. Different concentrations (5–25 mg/mL) of Mg(NO_3_)_2_-NPs were prepared in DMSO. The discs were soaked in the suspension and dried in a sterile environment at 80 °C.

### 2.9. Antibacterial Activity of Mg(NO_3_)_2_-NPs Coated Filter Discs Against MDR Bacteria

The antibacterial effects of the Mg(NO_3_)_2_-NPs-coated filter discs against MDR bacteria were assessed by the Kirby–Bauer disc diffusion method as recommended by CLSI [18]. MHA plates were inoculated with different MDR bacterial strains, and filter paper discs coated with nanoparticles were placed on them. The plates were incubated at 37 °C for 24 h. The diameter of the zone of inhibition was measured in millimetres. DMSO was used as the negative control, and antibiotic discs as the positive controls.

### 2.10. Determination of MIC and MBC of Mg(NO_3_)_2_-NPs

The MIC of Mg(NO_3_)_2_-NPs was measured by the broth microdilution method in sterile 96-well micro-titer plates according to the principles of antimicrobial susceptibility testing. In this assay, Mg(NO_3_)_2_-NPs were dispersed in sterile Mueller–Hinton broth and twofold diluted to get concentrations of 25, 12.5, 6.25, 3.125, 1.56, 0.78, 0.39 and 0.19 mg/mL. Fresh overnight cultures of *E. coli*, *S. aureus* and *Pseudomonas* spp. at approximately 0.5 McFarland standard were diluted to a final inoculum concentration of 5 × 10^5^ CFU/mL in each well. Broth and bacterial inoculum were used as growth control; broth only was used as sterility control, while DMSO was used as solvent control.

The plates were incubated at 37 °C for 24 h. The lowest concentration of nanoparticles at which no visible growth was observed was taken as the MIC. The MBC was determined by taking 10 µL of samples from wells with no visible growth and subculturing them on MHA plates, followed by incubation at 37 °C for 24 h. The MBC was recorded as having the lowest concentration with no colony formation on MHA plates. Both MIC and MBC experiments were conducted in triplicate.

### 2.11. Growth Kinetics Analysis of Mg(NO_3_)_2_-NPs

To assess the bactericidal and bacteriostatic activity of Mg(NO_3_)_2_-NPs against the MDR bacteria (*E. coli*, *S. aureus* and *Pseudomonas* spp.), the time-kill assay was conducted. Bacterial suspension was prepared in Mueller–Hinton broth and standardized to 10^6^ CFU/mL. The concentrations of the Mg(NO_3_)_2_-NPs used for each suspension were equal to 0.5×, 1×, and 2× MIC determined by broth microdilution according to CLSI guidelines. Cultures not treated but incubated under the same conditions were used as growth controls. All tubes were cultured for 24 h at 37 °C on an orbital shaker with shaking (150 rpm). A total of 100 µL samples were aseptically collected at regular intervals (0, 2, 4, 6, 8, 12 and 24 h), serially diluted in sterile phosphate-buffered saline (pH 7.4), and spot-plated on MHA plates in triplicate. After 18 to 24 h of incubation, colonies were counted and reported as log_10_ (CFU/mL). Bactericidal activity was a reduction in viable cell count of ≥3 log_10_ compared to the initial inoculum. Each experiment was repeated three times (*n* = 3), and results are presented as mean ± standard deviation (SD). The statistical analysis was carried out by applying one-way ANOVA with Tukey’s post hoc test (IBM SPSS Statistics, version 25) and a *p*-value < 0.05 was regarded as statistically significant.

### 2.12. Preparation of Nanoparticle-Coated Antibiotic Discs

A solution of 0.25 mg/mL of Mg(NO_3_)_2_-NPs was prepared in distilled water. A volume of 5 mL of the nanoparticle suspension was poured onto antibiotic discs and dried at 80 °C to produce nanoparticle-loaded antibiotic discs.

### 2.13. Comparative Antibacterial Assay for Mg(NO_3_)_2_-NPs Coated and Uncoated Antibiotics

The standard disc diffusion method was used to assess the antibacterial activity of nanoparticle-coated and uncoated antibiotic discs [18]. MHA plates were inoculated with bacteria, and discs were placed on the plates for 24 h at 37 °C. The diameter of the zones of inhibition was determined and compared to evaluate the activity of the antibiotic.

### 2.14. Toxicity Assessment Using Brine Shrimp Lethality Assay (BSLA)

The Brine Shrimp Lethality Assay (BSLA) was used to evaluate the preliminary toxicity of Mg(NO_3_)_2_-NPs. The eggs of *Artemia salina* were incubated in artificial seawater for 24–48 h with continuous light and aeration. The motile nauplii were transferred to test tube plates with 5 mL of artificial seawater. Mg(NO_3_)_2_-NPs were tested at concentrations of 25, 50, 100, 250, 500, and 1000 µg/mL. Five nauplii were added to each concentration. Artificial seawater was used as a negative control, and potassium dichromate was used as a positive control. Following 24 h incubation at room temperature, the mortality of nauplii was recorded. The percentage mortality was calculated as:

$$\mathrm{Mortality}\;(\%)=\frac{\mathrm{Number}\;\mathrm{of}\;\mathrm{dead}\;\mathrm{nauplii}}{\mathrm{Total}\;\mathrm{number}\;\mathrm{of}\;\mathrm{nauplii}}\times 100$$

The median lethal concentration (LC_50_) was determined by regression/probit analysis. The sample was considered practically non-toxic if the LC_50_ was greater than 1000 µg/mL in the preliminary screening procedure. The lethality of brine shrimp, with the calculation of LC_50_ values, was used as a rapid toxicity screening and the values were calculated based on mortality against log concentration after 24 h exposure.

### 2.15. Statistical Analysis

Each experiment was repeated three times, and the results were presented as mean ± standard error (SE). Data were analyzed using one-way analysis of variance (ANOVA) using statistical software (IBM SPSS Statistics, version 25). Data with *p*-values less than 0.05 were considered statistically significant data.

## 3. Results

### 3.1. Confirmation of MDR Bacterial Isolates

The collected MDR bacterial isolates (*E. coli*, *Pseudomonas* spp. and *S. aureus*) were successfully revived and identified by morphological and biochemical tests. Microscopic analysis confirmed that *E. coli* and *Pseudomonas* spp. had rod-shaped Gram-negative morphology while *S. aureus* had Gram-positive cocci in clusters, as expected from their classification.

Furthermore, biochemical tests confirmed their identification. Catalase activity was positive for all MDR bacterial isolates. *Pseudomonas* spp. was oxidase positive, while *E. coli* and *S. aureus* were oxidase negative. Both *Pseudomonas* spp. and *S. aureus* demonstrated the ability to use citrate, with *E. coli* being citrate negative. *E. coli* was indole positive, and *S. aureus* was positive for Voges-Proskauer (VP).

Antibiotic susceptibility testing showed all isolates were resistant to ceftazidime, penicillin, oxacillin and erythromycin antibiotics. This pattern of resistance to different classes of antibiotics confirmed the MDR status of the bacterial isolates (Table S1).

### 3.2. Phytochemical Screening of M. charantia Extracts

Phytochemical qualitative testing identified a variety of bioactive components in *M. charantia* extracts (Table 1). The methanolic extract contained tannins, flavonoids and terpenoids, while the aqueous extract mainly contained phenolics and saponins. The variation in phytochemical constituents of both extracts was due to the selective nature of the solvent used in extraction.

### 3.3. Antibacterial Activity of M. charantia Against MDR Bacteria

*M. charantia* extracts showed good antibacterial activity against all the tested MDR bacteria (Table 2). The methanolic extract was more active than the aqueous extract for all tested MDR bacteria. *S. aureus* was found to be the most susceptible isolate, with zones of 17.6 ± 0.23 mm and 15.3 ± 0.8 mm against methanolic and aqueous extracts, respectively. *Pseudomonas* spp. had an intermediate sensitivity, with zones of 16.2 ± 1.2 mm and 14.5 ± 0.5 mm for methanolic and aqueous extracts, respectively. *E. coli* was relatively less sensitive with zones of 13.3 ± 0.5 mm and 12.3 ± 0.3 mm against methanolic and aqueous extracts, respectively. This suggests greater susceptibility of Gram-positive bacteria to Gram-negative bacteria. The antibacterial activity was significant according to statistical analysis (*p* = 0.0032).

### 3.4. Characterization of Mg(NO_3_)_2_-NPs

#### 3.4.1. SEM Analysis

**Figure 1:**
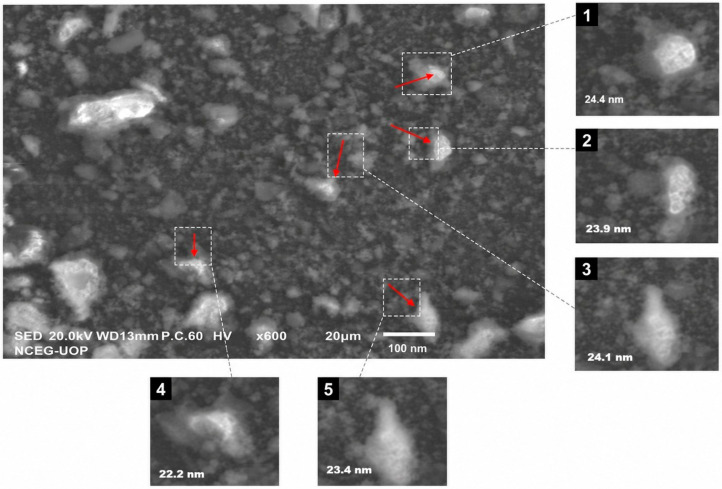
SEM image of biosynthesized Mg(NO_3_)_2_-NPs depicts abundance of irregular and multi-faceted nanoscale particles, which are polydisperse and heterogeneous. The particles are found in the shapes of quasi-spherical, rod-like, triangular, angular, and faced with localized agglomeration throughout the surface of the particles. The increase in the inset areas suggests representative particle sizes of 22–25 nm for the Mg(NO_3_)_2_-NPs, which correlates with the average crystallite size of 23.6 nm obtained from XRD, thereby concluding the formation of Mg(NO_3_)_2_-NPs at the nanoscale.

The SEM micrograph of the synthesized Mg(NO_3_)_2_-NPs revealed a polydisperse and heterogeneous morphology with partially agglomerated particles embedded in a fine granular matrix. The nanoparticles showed multi-faceted, irregular shapes such as angular, quasi-spherical, triangular, rod-shaped and faceted shapes, indicating anisotropic growth during the formation of nanoparticles. The enlarged regions selected displayed representative particle sizes of 22–25 nm, which is in good agreement with the average crystallite size, obtained from XRD analysis, of 23.6 nm. Localized agglomeration may be explained by the high surface energy of the particles and the forces between particles during drying. The observations are in good agreement with the successful formation of irregularly shaped nanoscale Mg(NO_3_)_2_-NPs and moderate aggregation (Figure 1).

#### 3.4.2. EDAX Analysis

**Figure 2:**
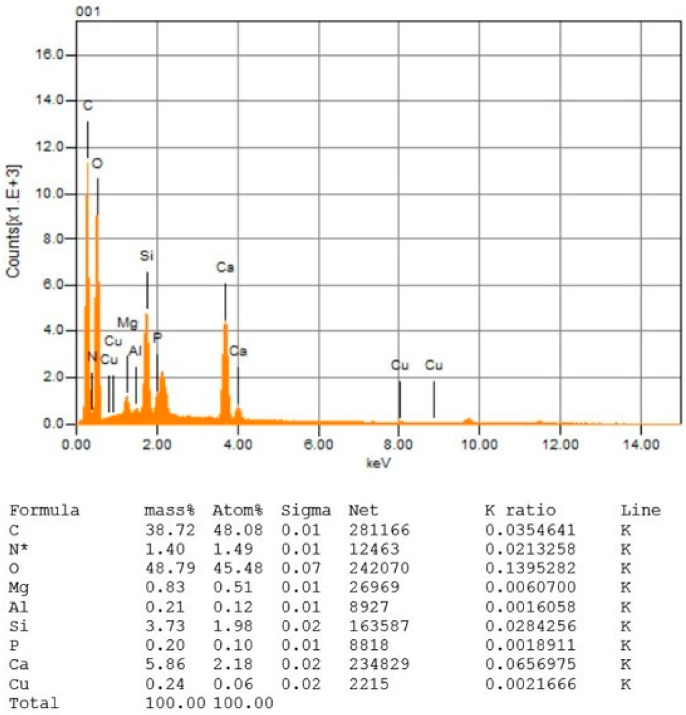
EDAX spectrum of biosynthesized Mg(NO_3_)_2_-NPs showing major peaks of Mg, N and O elements, confirming the formation of Mg nanostructures, and minor peaks for Si, Ca, Al, P, C, and Cu elements, suggesting trace elements and biomolecules from the plant extract.

The elemental composition of the nanoparticle was investigated by EDAX analysis. The EDAX spectrum showed strong peaks corresponding to magnesium (Mg), nitrogen (N) and oxygen (O) and indicated the formation of Mg-based nanoparticles. The presence of minor peaks for silicon (Si), calcium (Ca), phosphorus (P), aluminum (Al), carbon (C) and copper (Cu) were also detected. The oxygen and carbon peaks are probably due to the plant-derived organic material on the surface of the nanoparticles, while the other minor peaks may be due to the presence of other biomolecules and/or the background of the instruments (Figure 2).

#### 3.4.3. FTIR Analysis

The functional groups present in the synthesized Mg(NO_3_)_2_-NPs were identified using FTIR analysis, and the interaction of the plant biomolecules with the nanoparticles was studied to understand the mechanisms of their reduction, capping and stabilization. The FTIR spectrum revealed a broad band at 3260 cm^−1^, which was assigned to O-H stretching of alcohols or phenols. These groups are reported to act as initial reducing agents by donating electrons to the magnesium ions and then form a hydrogen bond to stabilize the nanoparticles. The peak at 2982 cm^−1^ was attributed to N-H stretching vibrations of amines or protein groups, which are involved in the stabilization of nanoparticles through coordination and surface adsorption.

The band at 2188 cm^−1^ corresponded to C≡C stretching of alkynes and could interact with the surface of the nanoparticles through weak interactions. The peak at 1639 cm^−1^ corresponded to aromatic C=C or C-H stretching, suggesting the presence of phenolic groups that can contribute to nanoparticle stability via π-π stacking and delocalization. The band at 1550 cm^−1^ was attributed to N-O stretching vibrations of nitro groups that can be involved in electrostatic stabilization of the nanoparticle surface. The peaks at 1327 cm^−1^ (carboxylic O-H) and 1203 cm^−1^ (C-F stretching) indicate the presence of organic acids and fluorinated compounds, respectively, which can serve as surface capping agents by interacting with nanoparticle surfaces and preventing aggregation.

**Figure 3:**
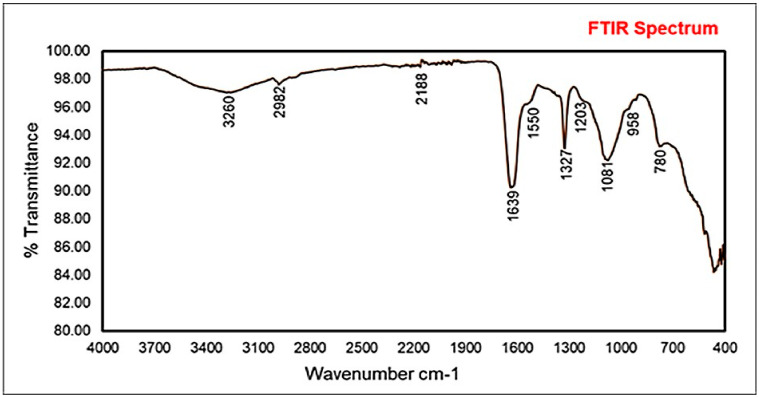
FTIR analysis of biosynthesized Mg(NO_3_)_2_-NPs showing characteristic absorption bands at 3260 cm^−1^ (O–H), 2982 cm^−1^ (N–H), 2188 cm^−1^ (C≡C), 1639 cm^−1^ (aromatic C–H/C=C), 1550 cm^−1^ (N–O), 1327 cm^−1^ (O–H of carboxylic acid), 1203 cm^−1^ (C–F), 1081 cm^−1^ (C–O), 958 cm^−1^ (C=C), and 780 cm^−1^ (alkene), indicating the involvement of various functional groups in reduction, capping, and stabilization of nanoparticles.

The peaks at 1081 cm^−1^ and 958 cm^−1^ indicated C-O stretching of alcohols and ethers, which are important for nanoparticle stabilization via interaction with metal ions. The band at 780 cm^−1^ (alkene C=C) also indicates the presence of unsaturated organic groups in nanoparticle stabilization. In summary, the FTIR analysis confirms the involvement of multiple phytoconstituents such as phenolics, proteins and organic acids in the reduction of Mg^2+^ ions and as natural capping and stabilizing agents, leading to the prevention of nanoparticle aggregation and improvement in stability (Figure 3; Table S2).

#### 3.4.4. XRD Analysis

The XRD technique was used to investigate the phase purity, crystallinity and particle size of the Mg(NO_3_)_2_-NPs. The XRD peaks were well-defined, suggesting that the nanoparticles were crystalline. These peaks correspond to different planes (*hkl*) of Mg(NO_3_)_2_-NPs. The peaks at (100), (002), (101), (102) and (110) planes were relatively higher, indicating a highly ordered crystal structure. The agreement of these peaks with the standard JCPDS data suggests the phase purity and successful preparation of Mg(NO_3_)_2_-NPs without noticeable impurities.

**Figure 4:**
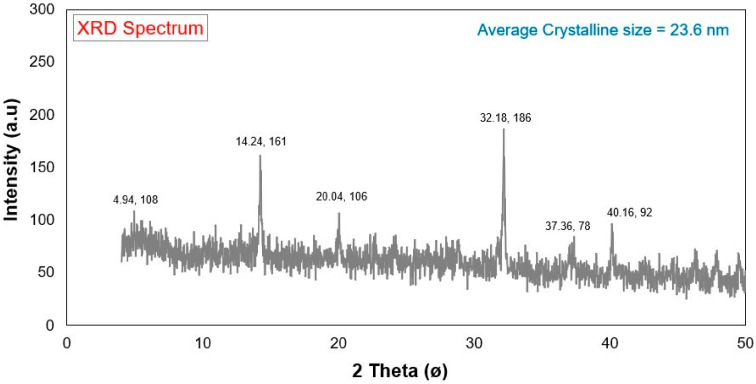
XRD pattern of biosynthesized Mg(NO_3_)_2_-NPs showing well-defined crystalline peaks, confirming the presence of planes (100), (002), (101), (102) and (110) in accordance with the standard JCPDS data, implying phase purity and crystalline nature, with an average crystallite size of 23.6 nm calculated by the Debye–Scherrer equation.

The crystallite size (D) of the nanoparticles was estimated by the Debye–Scherrer formula: $D=\frac{K\lambda }{\beta cos\theta }$, where D is the diameter of the crystallites, K is the shape factor (usually 0.9), λ is the wavelength of the Cu-Kα radiation (1.5406 Å), β is the full width at half maximum (FWHM) of the peak, and θ is the Bragg angle. The crystallite size of the synthesized Mg(NO_3_)_2_-NPs was calculated to be around 23.6 nm. This crystallite size agrees with the SEM analysis and suggests the formation of very fine crystallites. The small size of the crystallites implies a large surface-to-volume ratio, which is crucial for the nanoparticles’ improved physicochemical and biological properties. The intensity and sharpness of the peaks also suggest good crystallinity and stability of the nanoparticles (Figure 4).

### 3.5. Antibacterial Activity of Mg(NO_3_)_2_-NPs Coated Filter Paper Discs

Mg(NO_3_)_2_-NPs showed a concentration-dependent antibacterial activity against all MDR bacterial strains (Table 3). At the lowest concentration (5 mg/mL), low inhibition was observed, but increasing nanoparticle concentration resulted in a gradual increase in antibacterial effect. The highest inhibition was noted with the highest concentration (25 mg/mL) against *S. aureus* (17.6 ± 1.1 mm), followed by *E. coli* (14.03 ± 0.8 mm) and *Pseudomonas* spp. (8.6 ± 1.7 mm). This pattern reveals the increased antibacterial efficacy with increasing concentration of nanoparticles, suggesting a dose-dependent effect. A significant difference was observed between concentrations (*p* = 0.00013).

### 3.6. MIC and MBC of Mg(NO_3_)_2_-NPs Against MDR Bacteria

Mg(NO_3_)_2_-NPs demonstrated a quantitative inhibitory and bactericidal effect on the MDR bacteria (Table 4). The MIC of Mg(NO_3_)_2_-NPs was low against *S. aureus* (6.25 mg/mL), followed by *E. coli* (12.5 mg/mL) and *Pseudomonas* spp. (25 mg/mL). A comparable pattern was seen with the MBC, with *S. aureus* showing the lowest MBC. The results demonstrate the antibacterial potential of Mg(NO_3_)_2_-NPs in addition to the zone of inhibition.

### 3.7. Growth Kinetics of Mg(NO_3_)_2_-NPs Against MDR Bacteria

The antimicrobial response of MDR bacteria, such as *E. coli*, *S. aureus*, and *Pseudomonas* spp., is presented in the form of time-kill kinetics (Table 5) for each bacterium tested with various concentrations of Mg(NO_3_)_2_-NPs, which shows that the response from the bacteria is dose-dependent and strain-specific. Spontaneous bactericidal activity was not found in all untreated control cultures of *E. coli*, *S. aureus* and *Pseudomonas* spp. cultures, and a validation of the experimental baseline was established as all the cultures grew unimpeded in an exponential growth manner throughout 24 h of the experiment.

All three MDR bacteria showed a delayed onset of the exponential growth phase compared to the untreated controls, with a progressive reduction in viable bacteria after 0.5× MIC (*p* < 0.05) consistent with bacteriostatic activity. This sub-inhibitory dose did not meet the bactericidal criterion, but the lag period observed suggests that there is substantial antibacterial suppression at lower doses of nanoparticles for all the MDR bacteria.

*E. coli* showed a progressive decrease over 24 h at 1× MIC (bacteriostatic, *p* < 0.01). At 1× MIC, *S. aureus* was the most susceptible of all strains with near-complete growth inhibition after 8–12 h of exposure (*p* < 0.001) and was the most responsive strain. In contrast, *Pseudomonas* spp. demonstrated reduced, but ongoing bacterial proliferation over 24 h, suggesting partial tolerance to the inhibition by nanoparticles (*p* < 0.05) at this concentration.

A bactericidal concentration of 2× MIC was used for all bacteria, with *S. aureus* showing the fastest killing rates, reducing the bacterial load by at least 3 log_10_ of S. aureus in 6–8 h after exposure to the nanoparticles and complete killing after 24 h (*p* < 0.001). The *E. coli* also met the bactericidal criterion, achieving a ≥3 log_10_ CFU/mL reduction after 12 h of treatment and a complete kill after 24 h (*p* < 0.001). The findings indicate that the bactericidal activity of the Mg(NO_3_)_2_-NPs against both MDR Gram-positive and Gram-negative bacteria is clinically relevant within 24 h.

### 3.8. Antibacterial Effect of Mg(NO_3_)_2_-NPs Coated and Uncoated Antibiotic Against MDR Bacteria

The zone of inhibition of the antibiotics was significantly enhanced by the coating of Mg(NO_3_)_2_-NPs (Table 6). Antibiotic discs without nanoparticles produced no zones of inhibition against the MDR bacteria, thus confirming resistance. However, coated antibiotics exhibited zones of inhibition against all organisms.

The greatest enhancement in antibiotic’s potency after coating with Mg(NO_3_)_2_-NPs was seen with *S. aureus* (15.3 ± 0.3 mm–16.6 ± 0.5 mm), and comparable enhancements were seen with *E. coli* (13.4–14.3 mm) and *Pseudomonas* spp. (12.2–12.4 mm). This study shows a marked enhancement in antibiotic activity when coated with nanoparticles, suggesting a synergy between Mg(NO_3_)_2_-NPs and antibiotics (*p* = 0.0021).

### 3.9. Brine Shrimp Lethality Assay (BSLA) of Mg(NO_3_)_2_-NPs

**Figure 5:**
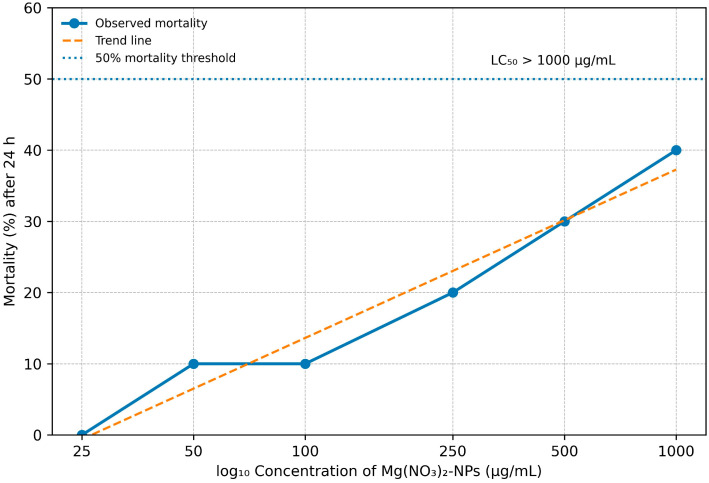
Dose–response curve of Mg(NO_3_)_2_-NPs that was measured using BSLA. The concentration of nanoparticles in the exposure of *A. salina* nauplii grew with time and with concentration, increasing the mortality of the organism. The trend of dose response was shallow, and the highest mortality was 40% at 1000 µg/mL. The lack of 50% lethality in the range of the experiment demonstrates the low acute toxicity, with the estimated LC_50_ higher than 1000 µg/mL.

The BSLA was used to study the toxicity of Mg(NO_3_)_2_-NPs. The percent mortality increased with increasing concentrations of nanoparticles, suggesting a dose-dependent effect. But mortality was low at concentrations up to 250 µg/mL, indicating good preliminary biocompatibility at low concentration. The LC_50_ value (deadly concentration at 50%) calculated was more than 1000 µg/mL, suggesting low acute toxicity in the brine shrimp model as shown in Figure 5 and Table S3.

## 4. Discussion

The MDR phenotypes of *E. coli*, *S. aureus* and *Pseudomonas* spp. in this study are like the well-described resistance mechanisms of these bacteria, which include drug target modification, drug inactivation, efflux and biofilm tolerance. These processes decrease the concentration of drugs and their efficacy in the cell [23]. All strains were completely resistant to multiple antibiotics, which indicates a very selective environment, and this is consistent with previous studies reporting the rapid dissemination of MDR phenotypes through horizontal gene transfer and plasmid transfer [24].

Based on the phytochemical study of *M. charantia* extracts, tannins, flavonoids and terpenoids were found in the methanolic extract, while phenolics and saponins were present in the aqueous extract. The selective extraction is due to the polarity and solubility of secondary metabolites. Flavonoids and phenolic compounds are known to have antimicrobial activity by disrupting cell membranes, chelating metal ions, blocking nucleic acid synthesis and disrupting energy production. The lipophilic properties of terpenoids allow them to penetrate the cell membrane, disrupting its integrity, leading to the leakage of cell contents. Previous studies have shown these effects to be antibacterial [25]. The chemical constituents in *M. charantia* peel extract, identified from qualitative screening (Table 1), specifically flavonoids, phenolics, tannins and terpenoids, have dual functional activities as both reducing and stabilizing agents in the green synthesis of Mg(NO_3_)_2_-NPs, which has been directly confirmed and explained by FTIR analysis (Figure 3, Table S2). Specifically, the hydroxyl (O-H) and phenolic groups identified in the FTIR directly correlated with the phenolic compounds identified in the phytochemical screening and act as electron donors to reduce the Mg^2+^ ion to produce metallic nanoparticles in the synthesis reaction. Amine and protein-derived groups were detected at 2982 cm^−1^, which are related to terpenoids and alkaloids and play a role in nucleation and initiation of nanoparticle synthesis. These same phytochemical components contribute to the stabilization of nanoparticles in several ways: hydroxyl and phenolic functionalities form hydrogen bonding with the surfaces of the nanoparticles; aromatic functional groups stabilize the nanoparticles through π-π stacking interactions; carboxylic acids (1327 cm^−1^) and amine (2982 cm^−1^) functional groups provide electrostatic stabilization; and C-O stretching vibrations (1081 cm^−1^) indicate the presence of ether linkages capable of coordinating with metal ions. Carboxylic acids and fluorinated compounds (1203 cm^−1^) detected in the FTIR are used as surface capping agents in the synthesis of nanoparticles, which build steric barriers to avoid aggregation and coalescence of nanoparticles. The role of the phytochemical-mediated stabilization mechanism is one of the reasons that plant-synthesized nanoparticles have superior biocompatibility to chemically synthesized nanoparticles, since the organic coating formed through plant phytochemicals prevents the interaction between the nanoparticles and cells without losing antimicrobial activity. The strong correlation of phytochemical classes with the identified functional groups (FTIR, Figure 3) and the resulting properties of the nanoparticles indicates that *M. charantia* peel extract is a multifunctional biological matrix that can simultaneously reduce the metal ions, initiate the formation of nanoparticles, stabilize metal nanoparticles from aggregation, and provide biocompatibility, which is the most comprehensive mechanism for green synthesis compared to a single-component chemical reducing agent.

The antibacterial activity of *M. charantia* extracts against MDR bacteria, with higher activity of methanolic extracts, agrees with the various phytochemicals extracted by methanol. The susceptibility of *S. aureus* to antimicrobial agents is different than that of the Gram-negative bacteria, which may be due to varying cell wall properties. Lipopolysaccharides constitute the outer membrane in Gram-negative bacteria, which hinders penetration of active molecules, but are not seen in Gram-positive bacteria. These results are consistent with the results of Jindal and Kaushalendra [26].

This study reveals the capacity of *M. charantia* peel extract to serve as a reducing, stabilizing and capping agent in the biosynthesis of Mg(NO_3_)_2_-NPs, indicating that plant phytochemicals play an active role in this process. The SEM micrograph showed a fine granular matrix with partially agglomerated and polydispersed nanoparticles distributed in it. The morphology of the particles deviated from a perfect spherical shape, with a more irregular and multi-faceted form, suggesting anisotropic nucleation and growth in the plant-mediated synthesis.

The enlarged SEM areas gave representative particle sizes in the range of 22–25 nm corresponding to the XRD-determined average crystallite size of 23.6 nm. This agreement validates that the synthesized Mg(NO_3_)_2_-NPs are at the nanoscale level. The variation in size and irregular morphology could be due to the preferential adsorption of phytochemicals like phenolic compounds, flavonoids and proteins on certain crystal planes, which would control directional growth. However, the observed localized agglomeration in the SEM image might be attributed to the high surface energy of nanoscale particles and interactions between the surfaces coated with the phytochemicals. The plant-mediated magnesium-based nanoparticles with similar morphological variations have been reported by Ahmad et al. [27] and Gatou et al. [28].

EDAX analysis has confirmed that nanoparticles are mainly composed of magnesium, nitrogen, carbon and oxygen. The presence of carbon and oxygen also indicates that the nanoparticles are capped by organic molecules. These organic capping agents stabilize the nanoparticles due to steric effects. This observation has been reported for the biosynthesis of nanoparticles using plant sources [29], where organic capping impacts nanoparticle stability and characteristics.

The crystallinity of nanoparticles, as confirmed by XRD (average crystallite size 23.6 nm), is an important factor in explaining the physical and biological behaviour of nanoparticles. Smaller crystallites have a higher surface/volume, thus a higher surface reactivity, and interaction with microorganisms. The smaller the size of the nanoparticles, the greater their penetration efficiency through the cell wall and membrane and the better their antimicrobial properties. The study reported by Ramezani-Farani et al. [29] indicates that the crystallite size of the magnesium nanoparticles is quite similar, and the reactivity is increased, which reflects the present study. Furthermore, the average crystallite size of 23.6 nm, obtained from XRD data, is quantitatively validated with the visual inspection of the nanoscale dimensions in the SEM micrograph. This XRD analysis is corroborated by the claimed particle size range of 1–100 nm based on SEM image visualization, and the calculated average crystallite size of 23.6 nm is within the wider range of the SEM visualization, confirming the nanoscale nature of the synthesized nanoparticles. The SEM morphological characterization (qualitative visual range of 1–100 nm) and the XRD crystallite sizing (quantitative average of 23.6 nm) offer complementary and comprehensive evidence for the successful synthesis of nanoparticles. Both qualitative SEM and quantitative XRD are used in this way as the standard approach for characterizing nanomaterials, and the Debye–Scherrer method is a well-established and internationally recognized method for determining dimensions of nanoparticles [29,30]. As a result, our XRD-based crystallographic analysis and SEM-based visual observations validate that Mg(NO_3_)_2_-NPs are indeed nanoscale.

FTIR spectra showed the presence of functional groups (hydroxyl, amine and aromatic groups), which implies the involvement of plant biomolecules in the formation of nanoparticles. They are groups that can donate electrons to reduce the Mg^2+^ ions and coordinate with the nanoparticles through coordination and adsorption. The role played by these functional groups in the dispersion of nanoparticles and in preventing nanoparticle aggregation to conserve biological activity is of great importance. Similar FTIR patterns have been reported by Al-Harbi et al. [30], which highlights the stabilizing properties of the phytochemicals on the nanoparticles.

The dose-dependent antibacterial activity of Mg(NO_3_)_2_-NPs suggests a concentration-dependent effect. The antibacterial action of magnesium nanoparticles may include disruption of the cell membrane, ROS formation, and interaction with biomolecules inside the cells, such as proteins and DNA. The greater susceptibility of *S. aureus* to Gram-negative bacteria reflects the weak cell wall of Gram-positive bacteria. Increasing nanoparticle concentration leads to higher surface interaction with bacteria, which results in cell membrane damage and cell death [31].

MIC and MBC are quantitative tests that are useful to supplement diffusion tests. In the present study, the MIC and MBC of Mg(NO_3_)_2_-NPs against *S. aureus* (Gram-positive) were lower than those of Gram-negative bacteria, which indicates higher sensitivity of Gram-positive bacteria. This finding is also in line with the reports of higher sensitivity of Gram-positive bacteria because of the absence of an outer membrane and hence higher penetration of nanoparticles [29,32]. In contrast, Gram-negative bacteria such as Pseudomonas spp. have an outer membrane (lipopolysaccharides) and efflux pumps, which reduce antibiotic penetration and enhance resistance [24]. The MBC/MIC ratio was also indicative of bactericidal activity, especially against the tested bacteria *S. aureus* and *E. coli*, and the MBC was two times lower than the MIC observed. Other studies have found similar bactericidal effects on magnesium nanoparticles, with greater reactivity and small size of the nanoparticles making it easier to interact with bacterial surfaces and inside bacterial cells [29]. The study validates the antibacterial ability of Mg(NO_3_)_2_-NPs, in addition to the studies on inhibition and proposes their use as an antimicrobial agent.

Bacterial growth kinetics give important information for the dynamic effect of antimicrobials. The present study showed that Mg(NO_3_)_2_-NPs exhibited a definite concentration-dependent antibacterial response against the MDR bacterial isolates, with progressive decreases in viable cell counts noted as the concentration of the Mg(NO_3_)_2_-NPs increased from 0.5× to 2× MIC. All isolates showed a significantly delayed onset of the exponential growth phase (*p* < 0.05) and gradual bacteriostatic pressure (*p* < 0.05) at sub-inhibitory concentrations (0.5× MIC), which represented a significant antibacterial pressure but not lethal effects. *S. aureus* was the most susceptible, producing near-total growth inhibition within 8–12 h (*p* < 0.001); *E. coli* gave a progressive growth inhibition that was bacteriostatic, and *Pseudomonas* spp. gave only partial growth inhibition and continued to grow through the 24 h period. A decrease of ≥3 log_10_ in CFU/mL, reflecting bactericidal activity, was achieved at 2x MIC; *S. aureus* had the lowest MIC, followed by *E. coli* (*p* < 0.001 for both strains) and *Pseudomonas* spp., which had the highest MIC. Similar time-kill curves have been found with metal-based nanoparticles, where a higher concentration leads to a more rapid killing of bacteria because of the increased damage to the bacterial membrane and induction of oxidative stress [32]. The inability of Mg(NO_3_)_2_-NPs to attain bactericidal concentrations against *Pseudomonas* spp. at 2x MIC, show a log_10_ reduction that never exceeded the bactericidal threshold over 24 h. The relative tolerance of *Pseudomonas* spp. to Mg(NO_3_)_2_-NPs is due to several inter-related intrinsic resistance mechanisms inherent in Gram-negative bacteria. The higher MIC (25 mg/mL) than that of Gram-positive *S. aureus* (6.25 mg/mL) may be attributed to the lipopolysaccharide-rich outer membrane of Gram-negative *Pseudomonas* spp., which acts as a selective permeability barrier that slows down the diffusion and penetration of nanoparticles to the inner cell membrane [24]. Second, *Pseudomonas* spp. constitutively expresses resistance–nodulation–division (RND) type efflux pumps (*MexAB-OprM*, *MexCD-OprJ*) and ATP-binding cassette (ABC) transporters that actively extrude antimicrobial agents and potentially nanoparticles from the cytoplasm, reducing intracellular accumulation [24]. The time-kill kinetics data we compiled (Table 5) further support this active resistance mechanism: *S. aureus* was able to achieve near-complete growth inhibition within 8–12 h at 1× MIC, while *Pseudomonas* spp. showed only partial growth inhibition with subsequent bacterial proliferation during the 24 h exposure, which is indicative of continued efflux pump activity to overcome the stress induced by exposure to the nanoparticles. Third, under antimicrobial stress, *Pseudomonas* spp. can adaptively change the composition of LPS and the outer membrane charge density, which decreases the adhesion of nanoparticles and their uptake by cells [24].

Nanoparticles are active against bacteria through different mechanisms, including the production of ROS, denaturation of proteins and DNA replication, leading to a progressive decrease in cell viability [10]. Therefore, the growth kinetics results not only corroborate the MIC/MBC results but also reflect the dynamic interaction between nanoparticles and bacteria, including time- and concentration-dependent killing.

The other important aspect of this study is the remarkable improvement in the efficacy of antibiotics when coated with Mg(NO_3_)_2_-NPs. This synergistic effect can be attributed to the ability of nanoparticles to enhance the delivery of antibiotics and to bypass resistance mechanisms. Nanoparticles may increase the permeability of cell membranes, inhibit efflux pumps and penetrate biofilms, facilitating the entry of antibiotics into the cell. In addition, nanoparticles can be used as a delivery system for antibiotics. This combination effect has been reported by Salman et al. [31] and Das et al. [32], where nanoparticles and antibiotics were found to have a synergistic effect on the antibacterial activity against MDR bacteria. In addition, the *M. charantia* phytochemicals, including triterpenes, alkaloids and phenolics, may have a synergistic effect on the antibacterial effect of nanoparticles and antibiotics [14,33]. The synergistic effect of phytochemicals, nanoparticles and antibiotics is a multi-targeted approach to fight drug resistance.

It is necessary to determine the toxicity of nanoparticle-based antimicrobials to evaluate their potential biomedical applications. In the present study, Mg(NO_3_)_2_-NPs were nontoxic in the BSLA with an LC_50_ of over 1000 µg/mL, indicating good preliminary biocompatibility. BSLA has been extensively used as a rapid, cost-effective model for the assessment of acute toxicity of nanoparticles and has been shown to be a good predictor of cytotoxicity to higher organisms [34]. Recently, green-synthesized nanoparticles have shown low toxicity in the BSLA, and it has been concluded that the phytochemicals derived from the plant used for nanoparticle synthesis are responsible for the biocompatibility of the nanoparticles [35]. These natural surfactants protect against interactions between nanoparticles and cells and subsequent biological activity. In addition, nanoparticle toxicity has been reported to increase with an increase in concentration in *A. salina*, with higher mortality due to increased nanoparticle uptake and ROS generation [36]. This dose-dependent mortality at higher concentrations observed in the current study confirms the toxicity of Mg(NO_3_)_2_-NPs. However, low mortality at low concentrations suggests that the nanoparticles may selectively exert their antimicrobial property without being toxic. While BSLA has multiple benefits, it only provides preliminary toxicity data and further studies in mammalian cells and in vivo models must be conducted to establish the safety and therapeutic potential of these nanoparticles [37].

This study has some limitations. The in vitro analysis of antibacterial activity, including the MIC, MBC and growth kinetics, provides a quantitative and comprehensive result of the antibacterial effects of Mg(NO_3_)_2_-NPs, but it may not fully predict the response in the complex biological system. Growth kinetics revealed clear-time and dose-dependent killing effects, while more advanced mechanistic studies, such as ROS, membrane permeability and gene expression profiling, were required to elucidate the mechanism of action. The BSLA toxicity analysis indicated low toxicity and acceptable biocompatibility (at low concentrations) but only gives a preliminary estimate of the toxicity and may not predict toxicity in higher animals. Thus, additional studies with mammalian cell lines, in vivo studies and advanced mechanistic and toxicity studies are needed to confirm the clinical and therapeutic utility of Mg(NO_3_)_2_-NPs as an antibacterial and an adjuvant to antibiotics.

## 5. Conclusions

It was concluded that the green-synthesized Mg(NO_3_)_2_-NPs possess strong antibacterial activity, with *S. aureus* showing the lowest MIC (6.25 mg/mL), followed by *E. coli* and *Pseudomonas* spp. The comprehensive characterization revealed that the biosynthesized nanoparticles showed polydisperse morphology, tetrahedral, spherical and rod-like shapes ranging from 1 to 100 nm, high crystallinity (average crystallite size of 23.6 nm from XRD) and confirmed the phase purity by EDAX analysis. The presence of phytochemical functional groups such as phenolics, proteins and organic acids as reducing and capping agents was confirmed by FTIR analysis, which helped in stabilizing nanoparticles and preventing aggregation. The nanoparticles displayed bactericidal effects with concentration-dependent kinetics, using ≥3 log_10_ reduction with 2× MIC in 24 h. Interestingly, the efficacy of the conventional antibiotics was completely recovered by coating them with the Mg(NO_3_)_2_-NPs against resistance isolates, indicating a possible synergism in the therapeutic role. The preliminary toxicity testing in the BSLA (LC_50_ > 1000 µg/mL) suggests good biocompatibility at clinically relevant concentrations. But to prove the safety and therapeutic potential of such green-synthesized nanoparticles as a standalone antimicrobial agent or as an antibiotic adjuvant to combat MDR, more toxicity assessment in mammalian cell lines and in vivo infection models is necessary.

## Figures and Tables

**Table 1:** Phytochemical analysis of *M. charantia* aqueous and methanolic extracts.

Phytochemicals	Aqueous Extract	Methanolic Extract
Tannins	−	+++
Flavonoids	−	++
Terpenoids	−	+++
Phenolic compounds	+++	−
Saponins	+++	−

Note: +++ = Strong; ++ = Moderate; − = Absent.

**Table 2:** Antibacterial activity of *M. charantia* extracts against MDR bacteria.

S. NO.	MDR Bacteria	Activity of Methanolic Extract in mm	Activity of Aqueous Extract in mm	Positive Control—Penicillin	Negative Control—DMSO (1%)	*p* Value
1	*Pseudomonas* spp.	16.2 ± 1.2	14.5 ± 0.5	R	–	0.0032
2	*E. coli*	13.3 ± 0.5	12.3 ± 0.3	R	–	
3	*S. aureus*	17.6 ± 0.23	15.3 ± 0.8	R	–	

Note: Antibacterial activity of *M. charantia* extracts was evaluated using the agar well diffusion assay against MDR bacterial isolates. Methanolic and aqueous extracts were tested, and antibacterial activity was expressed as the zone of inhibition (mm). Penicillin was used as the positive control, while 1% DMSO served as the negative control. Values represent the mean zone of inhibition (mm) ± standard deviation (SD) from three independent replicates (*n* = 3). Statistical significance was determined using one-way ANOVA, and the corresponding *p*-value was provided where applicable. CLSI interpretations were applied for the positive control, where “R” indicates resistance of the tested isolates to penicillin.

**Table 3:** Antibacterial activity of Mg(NO_3_)_2_-NPs-coated filter paper discs against MDR bacteria.

S. NO.	MDR Bacteria	Antibacterial Activity of Mg(NO_3_)_2_-NPs Coated Filter Paper Discs Against MDR Bacteria in mm					Positive Control—Penicillin	Negative Control—DMSO (1%)	*p* Value
		5 mg/mL	10 mg/mL	15 mg/mL	20 mg/mL	25 mg/mL			
1	*Pseudomonas* spp.	6.1 ± 0.4	6.9 ± 1.5	7.3 ± 0.5	8.1 ± 1.3	8.6 ± 1.7	R	–	0.00013
2	*E. coli*	6.4 ± 1.3	7.2 ± 0.6	9.3 ± 1.2	11.3 ± 0.8	14.03 ± 0.8	R	–	
3	*S. aureus*	6.7 ± 0.5	7.7 ± 0.6	9.65 ± 0.8	13.4 ± 0.7	17.6 ± 1.1	R	–	

Note: Antibacterial activity of Mg(NO_3_)_2_-NPs coated on filter paper discs was evaluated using the disc diffusion method against MDR bacterial isolates. Nanoparticles were tested at concentrations of 5–25 mg/mL, and antibacterial activity was expressed as the zone of inhibition (mm). Penicillin was used as the positive control, while 1% DMSO served as the negative control. Values represent the mean zone of inhibition (mm) ± standard deviation (SD) from three independent replicates (*n* = 3). Statistical significance was determined using one-way ANOVA, and corresponding *p*-values were provided where applicable. CLSI interpretations were applied for the positive control, where “R” indicates resistance of the tested isolates to penicillin.

**Table 4:** MIC and MBC values of Mg(NO_3_)_2_-NPs against MDR bacterial isolates.

MDR Bacteria	MIC of Mg(NO_3_)_2_-NPs	MBC of Mg(NO_3_)_2_-NPs	MBC/MIC Ratio	Interpretation
*Pseudomonas* spp.	25 mg/mL	>25 mg/mL	>1	Weak bactericidal effect
*E. coli*	12.5 mg/mL	25 mg/mL	2	Bactericidal tendency
*S. aureus*	6.25 mg/mL	12.5 mg/mL	2	Strongest antibacterial response

Note: The MIC/MBC results supported the disc diffusion findings, where *S. aureus* showed the highest susceptibility to Mg(NO_3_)_2_-NPs, while *Pseudomonas* spp. showed comparatively lower sensitivity. This pattern may be attributed to the structural differences between Gram-positive and Gram-negative bacterial envelopes.

**Table 5:** Log_10_ CFU/mL of MDR bacteria treated with Mg(NO_3_)_2_-NPs at different levels of MIC.

Bacterial Isolates	MIC Conc.	Log_10_ CFU/mL of Bacteria Treated with Mg(NO_3_)_2_-NPs at Different Time Intervals in h							*p*-Value (vs. Control)
		0 h	2 h	4 h	6 h	8 h	12 h	24 h	
*E. coli*	Control	6.00 ± 0.15	6.30 ± 0.18	6.70 ± 0.20	7.20 ± 0.22	7.80 ± 0.25	8.50 ± 0.28	9.00 ± 0.30	—
	0.5× MIC	6.00 ± 0.15	6.10 ± 0.16	6.20 ± 0.17	6.40 ± 0.18	6.60 ± 0.19	7.00 ± 0.20	7.20 ± 0.21	<0.05
	1× MIC	6.00 ± 0.15	5.90 ± 0.17	5.70 ± 0.19	5.40 ± 0.21	5.10 ± 0.22	4.80 ± 0.24	4.50 ± 0.25	<0.01
	2× MIC	6.00 ± 0.15	5.80 ± 0.18	5.40 ± 0.20	4.80 ± 0.22	4.20 ± 0.24	3.50 ± 0.26	3.00 ± 0.28	<0.001
*S. aureus*	Control	6.00 ± 0.15	6.30 ± 0.18	6.70 ± 0.20	7.20 ± 0.22	7.80 ± 0.25	8.50 ± 0.28	9.00 ± 0.30	—
	0.5× MIC	6.00 ± 0.15	6.05 ± 0.16	6.10 ± 0.16	6.20 ± 0.17	6.30 ± 0.18	6.40 ± 0.18	6.50 ± 0.19	<0.05
	1× MIC	6.00 ± 0.15	5.80 ± 0.17	5.50 ± 0.19	5.10 ± 0.20	4.60 ± 0.22	3.80 ± 0.24	3.20 ± 0.25	<0.001
	2× MIC	6.00 ± 0.15	5.70 ± 0.18	5.20 ± 0.20	4.50 ± 0.22	3.80 ± 0.24	3.20 ± 0.25	2.80 ± 0.26	<0.001
*Pseudomonas* spp.	Control	6.00 ± 0.15	6.30 ± 0.18	6.70 ± 0.20	7.20 ± 0.22	7.80 ± 0.25	8.50 ± 0.28	9.00 ± 0.30	—
	0.5× MIC	6.00 ± 0.15	6.10 ± 0.16	6.25 ± 0.18	6.35 ± 0.19	6.50 ± 0.20	6.70 ± 0.21	6.90 ± 0.22	<0.05
	1× MIC	6.00 ± 0.15	6.00 ± 0.15	6.05 ± 0.16	6.10 ± 0.17	6.20 ± 0.18	6.40 ± 0.19	6.60 ± 0.20	<0.05
	2× MIC	6.00 ± 0.15	5.98 ± 0.16	5.95 ± 0.17	5.88 ± 0.18	5.80 ± 0.19	5.65 ± 0.20	5.40 ± 0.21	<0.05

Note: CFU = colony-forming units; MIC = minimum inhibitory concentration; ± = standard deviation. *p*-values derived from one-way ANOVA with Tukey’s post hoc test (GraphPad Prism v9.0) comparing each treatment group to untreated control at each time point.

**Table 6:** Antibacterial activity of Mg(NO_3_)_2_-NPs-coated and uncoated antibiotic discs against MDR bacteria.

MDR Bacteria	Antibacterial Activity of Mg(NO_3_)_2_-NPs Coated and Uncoated Antibiotic Discs Against MDR Bacteria								*p* Value
	Ceftazidime		Erythromycin		Oxacillin		Penicillin		
	Coated Disc	Uncoated Disc	Coated Disc	Uncoated Disc	Coated Disc	Uncoated Disc	Coated Disc	Uncoated Disc	
*Pseudomonas* spp.	12.2 ± 1.2	R	12.4 ± 0.7	R	12.2 ± 0.8	R	12.3 ± 0.8	R	0.0021
*E. coli*	14.1 ± 0.2	R	14.2 ± 0.9	R	13.4 ± 0.4	R	14.3 ± 0.5	R	
*S. aureus*	16.3 ± 0.7	R	16.6 ± 0.5	R	15.3 ± 0.3	R	15.4 ± 0.2	R	

Note: Antibacterial activity of Mg(NO_3_)_2_-NPs-coated and uncoated antibiotic discs was evaluated using the disc diffusion method against MDR bacterial isolates. Antibiotics tested included ceftazidime, erythromycin, oxacillin, and penicillin. Zones of inhibition (mm) were recorded for nanoparticle-coated and non-coated discs. Values represent the mean zone of inhibition (mm) ± standard deviation (SD) from three independent replicates (*n* = 3). Statistical significance was determined using one-way ANOVA, and the corresponding *p*-value was provided where applicable. CLSI interpretations were applied, where “R” indicates resistance of the tested isolates to the respective non-coated antibiotics. Enhanced activity observed in nanoparticle-coated discs suggests a potential synergistic effect between Mg(NO_3_)_2_-NPs and conventional antibiotics.

## Data Availability

All data supporting the findings of this study are included within the article. Further details are available from the corresponding author upon reasonable request.

## Supplementary Materials

The following supporting information can be downloaded at: https://www.mdpi.com/article/10.3390/nano16120728/s1, Table S1: Confirmation of Multidrug-Resistant (MDR) Bacterial Isolates; Table S2: FTIR Analysis of Biosynthesized Mg(NO_3_)_2_-NPs; Table S3: Brine Shrimp Lethality Assay (BSLA) Data for Mg(NO_3_)_2_-NPs.
