# Supplementary material for: Green Synthesis of Magnesium Nitrate Nanoparticles Using Momordica charantia Peel Extract: Enhanced Antibacterial Activity and Antibiotic Potentiation Against Multidrug-Resistant Bacteria with Low Acute Toxicity
Source: Nanomaterials (Basel). 2026 Jun 12;16(12):728. doi: 10.3390/nano16120728 (PMC13305694; doi:10.3390/nano16120728)
Supplement: Supplementary file 1 [file nanomaterials-16-00728-s001.zip › nanomaterials-4352880-supplementary.pdf]

Supplementary Materials

Green Synthesis of Magnesium Nitrate Nanoparticles using *Momordica charantia* Peel Extract: Enhanced antibacterial activity and antibiotic potentiation against multidrug-resistant bacteria with low acute toxicity

Table S1: Confirmation of Multidrug-Resistant (MDR) Bacterial Isolates.

S. No.	Bacterial Isolate	Gram Staining	Morphology	Key Biochemical Tests	MDR Confirmation
1	*E. coli*	Gram-negative	Rod-shaped	Oxidase−, Catalase+, Indole+, Citrate−, TSI: A/A	Resistant to ceftazidime, penicillin, oxacillin, erythromycin
2	*S. aureus*	Gram-positive	Cocci in clusters	Oxidase−, Catalase+, Citrate+, VP+, TSI: A/A	Resistant to ceftazidime, penicillin, oxacillin, erythromycin
3	*Pseudomonas* spp.	Gram-negative	Rod-shaped	Oxidase+, Catalase+, Citrate+, TSI: K/A	Resistant to ceftazidime, penicillin, oxacillin, erythromycin

Note: TSI = Triple Sugar Iron test; VP = Voges-Proskauer test; A = Acid production; K = Alkaline. All isolates demonstrated resistance to multiple antibiotics (four antibiotic classes), confirming their multidrug-resistant (MDR) phenotype.

Table S2: FTIR Analysis of Biosynthesized $Mg(NO_3)_2$-NPs.

Wavenumber (cm^{-1})	Functional Group	Interpretation
3260	O–H stretching	Alcohols/phenols; initial reducing agents
2982	N–H stretching	Amine/protein groups; stabilization through coordination
2188	C≡C stretching	Alkynes; weak interactions with nanoparticle surface
1639	Aromatic C–H/C=C	Phenolic groups; π–π stacking and delocalization
1550	N–O stretching	Nitro groups; electrostatic stabilization
1327	O–H of carboxylic acid	Organic acids; surface capping agents
1203	C–F stretching	Fluorinated compounds; surface capping
1081	C–O stretching	Alcohols/ethers; stabilization via metal ion interaction
958	C=C stretching	Unsaturated organic groups; stabilization
780	Alkene C=C	Unsaturated groups; nanoparticle stabilization
400–4000	Overall spectrum	Multiple functional groups involved in reduction, capping, and stabilization

Note: FTIR spectrum shows characteristic absorption bands confirming the involvement of plant biomolecules in the reduction, capping, and stabilization of $Mg(NO_3)_2$-NPs. The presence of multiple functional groups indicates that phytochemicals from *M. charantia* peel extract act as both reducing and capping agents.

Table S3: Brine Shrimp Lethality Assay (BSLA) Data for $Mg(NO_3)_2$-NPs.

Concentration (μg/mL)	Number of Nauplii	Number of Dead Nauplii	Mortality (%)
25	5	0	0
50	5	0	0
100	5	1	20
250	5	1	20
500	5	2	40
1000	5	2	40
LC_{50} (μg/mL)	—	—	>1000 (Non-toxic)

Note: BSLA was performed using *Artemia salina* nauplii. $Mg(NO_3)_2$-NPs showed dose-dependent mortality with an LC_{50} value greater than 1000 μg/mL, indicating low acute toxicity and acceptable biocompatibility at clinically relevant concentrations. Low mortality at concentrations up to 250 μg/mL suggests selective antimicrobial activity without toxicity at therapeutic doses.
